# Bone mesenchymal stem cell-derived extracellular vesicles deliver microRNA-23b to alleviate spinal cord injury by targeting toll-like receptor TLR4 and inhibiting NF-κB pathway activation

**DOI:** 10.1080/21655979.2021.1977562

**Published:** 2021-10-18

**Authors:** Hongfei Nie, Zhensong Jiang

**Affiliations:** a Department of Pain Management, West China Hospital of Sichuan University, Chengdu Sichuan, China; b Department of Spine Surgery, Shandong Provincial Hospital Affiliated to Shandong First Medical University, Jinan Shandong, China

**Keywords:** Spinal cord injury, bone mesenchymal stem cell, extracellular vesicles, miR-23b, tlr4/nf-κB, microglia

## Abstract

Bone mesenchymal stem cell-derived extracellular vesicles (BMSC-EVs) are known for recovery of injured tissues. We investigated the possible mechanism of BMSC-EVs in spinal cord injury (SCI). EVs were isolated from BMSCs and injected into SCI rats to evaluate the recovery of hindlimb motor function. The spinal cord tissue was stained after modeling to analyze spinal cord structure and inflammatory cell infiltration and detect microRNA (miR)-23b expression. The activity of lipopolysaccharide (LPS)-induced BV2 inflammatory cells was detected. The protein contents of interleukin (IL)-6, IL-1β, IL-10 and tumor necrosis factor-α (TNF-α) in spinal cord and BV2 cells were measured. Western blot analysis was used to detect the level of toll-like receptor (TLR)4, p65, p-p65, iNOS, and Arg1 in spinal cord tissue and cells. TLR4 was overexpressed in rats and cells to evaluate the content of inflammatory cytokines. After EV treatment, the motor function of SCI rats was improved, SCI was relieved, and miR-23b expression was increased. After treatment with EV-miR-23b, iNOS, IL-6, IL-1β, and TNF-α contents were decreased, while Arg1 and IL-10 were increased. The levels of TLR4 and p-p65 in spinal cord and BV2 cells were decreased. The rescue experiments verified that after overexpression of TLR4, the activity of BV2 cells was decreased, the contents of IL-6, IL-1β, TNF-α, and p-p65 were increased, IL-10 was decreased, and SCI was aggravated. To conclude, The miR-23b delivered by BMSC-EVs targets TLR4 and inhibits the activation of NF-κB pathway, relieves the inflammatory response, so as to improve SCI in rats.

## 1. Introduction

Spinal cord injury (SCI) is a life-changing injury in the central nervous system (CNS), which contributes to functional loss due to degenerative events including cell death and axonal damage [1–3]. More than one million patients around the world are paralyzed by SCI [4]. During the chronic stage, SCI results in severe deficits in motor, sensory and autonomic functions, thus affecting physical and mental health of SCI patients [5] and complex and long-term rehabilitation is required [6,7]. Apart from local injury within the spinal cord, SCI patients develop various organ dysfunctions and have increased susceptibility to pathogen infection, which hinder functional recovery and can even be devastating [8]. Neuroinflammation is increasingly recognized as a key pathophysiological mechanism in chronic neurodegeneration following SCI [9]. Therefore, it is crucial to develop a new therapy for SCI in terms of anti-inflammation.

Bone mesenchymal stem cells (BMSCs) are attractive to regenerative medicines due to their abilities in self-renewal, regeneration of damaged tissues and multilineage differentiation, and immunosuppressive capacity in regulating autoimmune diseases [10]. Transplantation of BMSCs to repair SCI has shown consistent benefits in preclinical models [11]. Importantly, MSCs could secrete several kinds of extracellular vesicles (EVs) to keep tissue homeostasis [12] and treat neurological and neurodegenerative conditions due to their anti-inflammatory and neuroprotective properties [13]. Administration of BMSC-EVs can reduce brain cell death, enhance neuronal survival and improve motor function [14]. EVs released from MSCs could attenuate apoptosis, inflammation, scarring activities and promote angiogenesis following SCI and facilitate functional recovery in SCI mice [15,16]. EVs contain mRNA, microRNAs (miRNAs), lipids and proteins and impact intercellular communication during normal physiology and pathological processes [17,18]. In addition, exosomal miRNAs are pivotal components in intercellular communication and cancer initiation and progression, and disease repair like SCI [19,20]. miRNAs may be potential targets for SCI treatment, modifying the processes of inflammation, apoptosis and functional recovery [21]. According to the literature, about 77% of mature miRNAs are expressed in the adult rat spinal cord. After SCI, the expression of nearly 300 miRNAs in the adult rat spinal cord has changed [22–24]. miR-23b regulates the NF-κB pathway to protect BV2 cells from apoptosis [25]. Additionally, miR-23b plays an important role in improving neuropathic pain of spinal cord injury [26]. But whether miR-23b is involved in the protective effects of BMSC-EVs on SCI is largely unknown. Based on the above references, we speculate whether miR-23b mediated by BMSC-EVs can regulate TLR4/NFκB signaling pathway, and then participate in the improvement of SCI in rats. Therefore, we performed a series of molecular and histochemical experiments in the established *in vivo* and *in vitro* models to investigate the mechanisms of BMSCs-EVs-mediated miR-23b in SCI via the TLR4/NF-κB pathway.

## 2. Materials and methods

### 2.1 Ethics statement

The study was conducted with the approval of the Ethical Committee of Shandong Provincial Hospital Affiliated to Shandong First Medical University (NO. HX2019072001). All experimental procedures were implemented on the Ethical Guidelines for the Study of Experimental Pain in Conscious Animals.

### 2.2 Isolation and identification of BMSCs

Specific pathogen-free male Sprague-Dawley (SD) rats (80–100 g, Hunan SJA Laboratory Animal Co., Ltd., Changsha, Hunan, China, animal license No. SYXK (Hunan) 2016–0002) were euthanized by an intraperitoneal injection of pentobarbital sodium (800 mg/kg), and the femur and tibia were removed under sterile conditions. Bone marrow buffer was collected with low-glucose Dulbecco’ s modified Eagle medium (L-DMEM) (Gibco, Grand Island, NY, USA) containing 10% fetal bovine serum (FBS; Gibco) and 1% penicillin–streptomycin (Solarbio Science & Technology Co., Beijing, China). The collected single cell suspension was centrifuged at 250 g for 5 minutes, then the supernatant was discarded, and cells were resuspended and seeded at 1 × 10^9^ cells/L. The medium was refreshed every three days. When the cell confluence reached 80–90%, the BMSCs were passaged and the cells at passage 3 (P3) were used for subsequent experiments. The morphology of P3 cells was observed under the microscope. Alizarin red (G1450, Solarbio), oil red O (G1262, Solarbio), and alcian blue (G2542, Solarbio) were utilized to identify the osteogenic, lipogenic, and chondrogenic differentiation characteristics of BMSCs. CD44-FITC (Cat#203,906), CD90-PE (Cat#205,903), CD34-PE (Cat#202,812), and CD-45-FITC (Cat#202,205) (all from eBiolegend, San Diego, CA, USA) were used for detection of the expression of CD44, CD90, CD34 and CD45 on the surface of BMSCs [27].

### 2.3 Isolation of BMSC-EVs

When the confluence reached 80%, BMSCs were washed with phosphate-buffered saline (PBS) three times, and the medium was replaced with the culture medium without EV serum. After 48 hours of culture, the supernatant was collected and centrifuged at 3000 g for 15 minutes to remove cells and cell fragments. The supernatant was centrifuged at 4°C for 30 minutes at 10,000 g, and filtered with 0.22 μm membrane (Steritop ™ Millipore, Burlington, MA, USA). After that, the supernatant was centrifuged at 4°C for 70 minutes and then discarded. The collected EVs were resuspended by PBS, and then centrifuged at 4°C for 70 minutes. Then obtained EVs were resuspended by 100 μL PBS and the protein concentration of EVs was quantified using a bicinchoninic acid (BCA) kit (Solarbio). The EVs were added to the complete culture medium and the concentration was adjusted to 200 μg/mL. The EVs were characterized and observed by transmission electron microscope (TEM/JEOL, Japan) and qNano system (Izon Science Ltd, New Zealand). The EV markers CD63, CD81, CD9 and Calnexin were examined by Western blot analysis [28]. At the same time, the GW group (BMSCs were cultured in the medium free of EV serum and treated with 10 μM GW4689 for 48 hours, and then BMSC medium supernatant was extracted) was set up.

miR-23b mimic and the negative control (NC) were provided by GenePharma (Suzhou, China). According to the instructions of Lipofectamine 3000 (Invitrogen, USA), miR-23b mimic and its NC were transfected into BMSCs, respectively, and named BMSCs-miR-23b and BMSCs-miR-con groups. After 48 hours of transfection, EVs were separated and extracted by the same method and named EVs-miR-23b group and EVs-miR-con group.

### 2.4 Establishment of SCI in rats

SD rats (N = 168) were raised at 20–26°C, provided with free diet and drinking water, and maintained under 12 h light/dark cycles. After adaptive feeding, rats (180–220 g) were randomly assigned into sham group (N = 24, injected with 0.5 mL PBS via the caudal vein), SCI group (N = 24, injected with 0.5 mL PBS via the caudal vein), SCI + GW group (N = 24, injected with 0.5 mL supernatant of BMSCs culture medium after intervention of GW4869 via the caudal vein), SCI + EVs group (N = 24, injected with 0.5 mL EVs with 100 μg EV protein via the caudal vein), SCI + EVs-miR-con (N = 24, 0.5 mL EVs-miR-con with 100 μg EV protein via the caudal vein), SCI + EVs-miR-23b (N = 24, 0.5 mL EVs-miR-23b with 100 μg EV protein via the caudal vein), SCI+Evs-miR-23b+ov-NC (N = 12, 0.5 mL EVs-miR-23b with 100 μg EV protein and 50 μL ov-NC via the caudal vein), and SCI+Evs-miR-23b+ov-TLR4 (N = 12, 0.5 mL EVs-miR-23b with 100 μg EV protein and 50 μL ov-TLR4 (2 × 10^9^ pfu) via the caudal vein) [28,29].

The rats were anesthetized by 2% sodium pentobarbital (40 mg/kg.bw, ip). The rats in the sham group underwent sham operation (only T9-T11 laminectomy), all rats in other groups underwent laminectomy in T9-T11 segment, and the whole spinal cord was transected with microsurgical scissors after lifting T10 segment of spinal cord with a spinal cord hook [30]. After model establishment, muscle and skin were sutured in layers, and artificial micturition was performed twice a day until autonomous micturition was recovered. At 24 hours after trauma, the rats were injected with EVs or GW via the tail vein [29,31]. On the 7^th^ or 28^th^ days after modeling, 12 rats were euthanized (treated with 100 mg/kg pentobarbital sodium) and the injured spinal cord samples in each group were collected for index detection. The samples of 6 rats were used for Nissl, hematoxylin and eosin (HE) staining and immunofluorescence staining, and the samples of remaining 6 rats were used for RT-qPCR, WB and ELISA. The time point of index detection and the number of animals required are shown in Table 1.

**Table 1.** The time point of index detection and the number of animals

Experiment	Animals amount of each group
Behavioral tests	N = 24/12 (before and 1, 3, 7, 14, 21 and 28 days after modeling)
Extraction of mRNA and protein	N = 6 (the 7^th^ day after surgery)
Nissl & HE &	N = 6 (the 7^th^ and 28^th^ day after surgery)
Immunofluorescence staining	N = 6 (the 7^th^ and 28^th^ day after surgery)

### 2.5 Evaluation of motor function of SCI rats

According to the previously described methods [32,33], Basso Beattie Bresnahan (BBB) score was employed to assess the recovery of hindlimb motor function of SCI rats before and 1, 3, 7, 14, 21 and 28 days after modeling. During the test, the rats were placed in open field for 5 minutes to move freely. The motor function of the rats was scored by two observers who were not experimenters but familiar with BBB quantitative score using a double-blind method. The average score of the two observers was used as the motor function score. In general, 0 points indicated complete paralysis and 21 points indicated normal movement. Scores between 1 and 20 indicated the corresponding motor function level of the hindlimbs of rats.

### 2.6 HE staining

The collected spinal cord samples at the 7^th^ and 28^th^ day after SCI surgery were fixed with 4% paraformaldehyde solution. After dehydration, xylene cleaning, wax immersion, paraffin embedding, sectioning (5 μm), dewaxing, dehydration, the sections were stained with HE staining kit (Solarbio), followed by dehydration using gradient ethanol, xylene cleaning, and neutral resin sealing. The sections were observed under the CX31 biomicroscope (Olympus, Japan) [27].

### 2.7 Nissl staining

The paraffined sections of spinal cord samples at the 7^th^ and 28^th^ day after SCI surgery were sliced and stained with Nissl staining kit (Solarbio). Nissl staining was photographed under the light microscope and the number of Nissl-positive cells was calculated to quantitatively analyze the number of surviving neurons [34].

### 2.8 Cell grouping

BV2 cells purchased from the cell resource center of Shanghai Institute of life sciences, Chinese Academy of Sciences were cultured in the H-DMEM (Gibco) containing a combination nof 10% FBS and 1% penicillin-streptomycin at 37°C and 5% CO_2_. To explore the effect of Evs-miR-23b on BV2 cells, when cell confluence reached 80%, the cells were passaged and assigned into blank group (untreated BV2 cells), LPS group (BV2 cells only underwent LPS induction), GW group (BV2 cells were cultured with GW and then induced by LPS), Evs group (BV2 cells were cultured with EVs and then induced by LPS), Evs-miR-con group (BV2 cells were cultured with Evs-miR-con and then induced by LPS), and Evs-miR-23b group (BV2 cells were cultured with Evs-miR-23b and then induced by LPS). Except the blank group, the cells in each group were added with EVs or GW (200 μg/mL [35], and LPS (100 ng/mL) [36] at the same time, and the subsequent experiments were carried out after incubation for 24 hours. To verify the effect of TLR4 on BV2 cells, we infected BV2 cells with ov-TLR4 or ov-NC (1 × 10^8^ TU/mL) before LPS induction. The packaging and construction of the overexpression vector were performed by GenePharma (Shanghai, China). BV2 cells overexpressing TLR4 and its control were prepared. The established cell model was co-incubated with Evs-miR-23b, and the corresponding groups were intervened with LPS for 24 hours. The cells were assigned into ov-TLR4 + Evs-miR-23b group (BV2 cells were infected with ov-TLR4, cultured with Evs-miR-23b, and induced by LPS) and ov-NC + Evs-miR-23b group (BV2 cells were infected with ov-NC, cultured with Evs-miR-23b, and induced by LPS).

### 2.9. 3-(4,5-dimethylthiazol-2-yl)-2,5-diphenyltetrazolium bromide (MTT) assay

The activity of BV2 cells was examined with the MTT method. After the intervention of EVs for 24 hours, the original medium was removed, and cells were added with 100 μL medium containing MTT (5 mg/mL). After 4-hour incubation, the medium was removed and the sediment was dissolved using dimethyl sulfoxide. The absorbance at the wavelength of 490 nm was measured.

### 2.10 Dual-luciferase reporter gene assay

The binding sites of miR-23b and TLR4 were predicted via the Starbase (http://starbase.sysu.edu.cn). The complementary binding sequence and mutation sequence of miR-23b and TLR4 were amplified and cloned into pmiR-Glo luciferase vector (Promega, Madison, WI, USA) to construct wild-type plasmid (TLR4-WT) and mutant plasmid (TLR4-MUT), and construct TLR-NC at the same time. According to the instructions of Lipofectamine^TM^ 3000 (Invitrogen, Carlsbad, CA, USA), the constructed plasmids were cotransfected with mimic NC and miR-23b mimic (GenePharma) into HEK293T cells (Shanghai Institute of cell biochemistry, Chinese Academy of Sciences). Luciferase activity was detected 48 hours later.

### 2.11 Enzyme-linked immunosorbent assay (ELISA)

The levels of interleukin (IL)-6, IL-1β, IL-10 and tumor necrosis factor-α (TNF-α) in the supernatant of spinal cord homogenate (at the 7^th^ and 28^th^ day after SCI surgery) and BV2 cells (at 24 hours after LPS treatment) were examined using ELISA kits (R&D Systems, MN, USA).

### 2.12 Western blot analysis

The spinal cord tissues were ground in liquid nitrogen or BV2 cells were lysed in strong radio-Immunoprecipitation assay lysis buffer (Beyotime Biotechnology Co., Ltd, Shanghai, China). The total protein was extracted using a Nuclear and Cytoplasmic Protein Extraction kit (P0027, Beyotime). The protein concentration was determined using a BCA kit (Beyotime). Equal amount of proteins (30 μg) was separated using 8–12% sodium dodecyl sulfate-polyacrylamide gel electrophoresis, and transferred to polyvinylidene fluoride membranes (Bio-Rad, Hercules, CA, USA). Then the membranes were blocked with 5% nonfat milk, and incubated with primary antibodies rabbit anti-TLR4 (ab13867, 2 µg/mL, 90KDa), rabbit anti-p65 (ab16502, 0.5 µg/mL, 64KDa), rabbit anti-p-p65 (ab86299, 1:5000, 60KDa), rabbit anti-iNOS (ab15323, 1 µg/mL, 140KDa), rabbit anti-Arg1 (ab91279, 1.0 µg/mL, 37KDa), mouse anti-CD63 (ab108950, 1:1000, 26KDa), mouse anti-CD81(ab109201, 1:2000, 26KDa), rabbit anti-CD9 (ab92726, 1:2000, 25KDa), rabbit anti-Calnexin (ab22595, 1 µg/mL, 90KDa), rabbit anti-β-actin (ab115777, 1:200, 42KDa), and rabbit anti-Lamin B1 (ab16048; 0.1 µg/mL, 68KDa), followed by incubation with secondary antibodies [goat anti-mouse IgG (ZB5305) and goat anti-rabbit (ZB-5301); 1:5000, ZSGB-Bio Co., Ltd, Beijing, China]. The bands were exposed and gray value was analyzed using Image-Pro Plus 6.0 software (Media Cybernetics, Inc., Rockville, MD, USA), with β-actin as the internal reference for total protein and Lamin B1 as the internal reference for nucleoprotein.

### 2.13 Reverse transcription quantitative polymerase chain reaction (RT-qPCR)

Total RNA was extracted from spinal cord tissue that was ground in liquid nitrogen or BV2 cells by TRIzol reagent (Invitrogen), and reverse transcribed to cDNA using Prime-Script RT reagent kit (Takara, Dalian, China). According to the instructions of SYBR ® Premix Ex Taq^TM^ II (Takara), the qPCR was detected on ABI7900HT fast PCR real-time system (Applied Biosystems, Foster City, CA, USA). The relative mRNA expression was normalized to GAPDH (internal parameter of TLR4) or U6 (internal parameter of miR-23b). The data were analyzed based on the 2^−ΔΔCT^ method. The amplified primer sequences of each gene and its primers are shown in Table 2.

**Table 2.** Primer sequences of RT-qPCR

Gene	Forward (5’-3’)	Reverse (5’-3’)
*TLR4*	ATGGCATGGCTTACACCACC	GAGGCCAATTTTGTCTCCACA
miR-23b	GAGGGTTCCTGGCATGC	GTGCAGGGTCCGAGGT
U6	TTCTTGGGTAGTTTGCAGTT	TTCTTGGGTAGTTTGCAGTT
*GAPDH*	GATTGTTGCCATCAACGACC	GTGCAGGATGCATTGCTGAC

### 2.14 Statistical analysis

Data analysis was introduced utilizing the SPSS 21.0 (IBM Corp., Armonk, NY, USA). Data are expressed as mean ± standard deviation. The *t* test was adopted for analysis of comparisons between two groups. One-way analysis of variance (ANOVA) was employed for the comparisons among multiple groups, and Tukey’s multiple comparison test was applied for the post hoc test after ANOVA. The *p* value was obtained from a two-tailed test, and *p* < 0.05 meant statistically significant.

## 3. Results

In this study, we aimed to explore the regulatory mechanism of bone marrow mesenchymal stem cell-derived extracellular vesicles (BMSCs-EVs)-mediated miR-23b in lipopolysaccharide (LPS)-induced spinal cord injury (SCI) *in vivo* and *in vitro* by negatively regulating the TLR4/NF-κB pathway. We established the LPS-induced SCI rat model and BV2 cell model, and successfully isolated (BMSCs-EVs). *In vivo*, we found that EVs could improve the activity of lower limbs, reduce tissue injury and inflammatory response, and upregulate the expression of mir-23b. EVs-mediated miR-23b (EVs-miR-23b) to participate in the differentiation of microglia after SCI and inhibit the occurrence of inflammatory response, improve the motor ability of SCI rats. *In vitro*, EVs-miR-23b inhibited the expression of inflammatory factors in BV2 cells, inhibit the expression of TLR4 and then inhibit the activation of NF-KB pathway.

### 3.1 Identification of BMSC-EV

**Figure 1.**
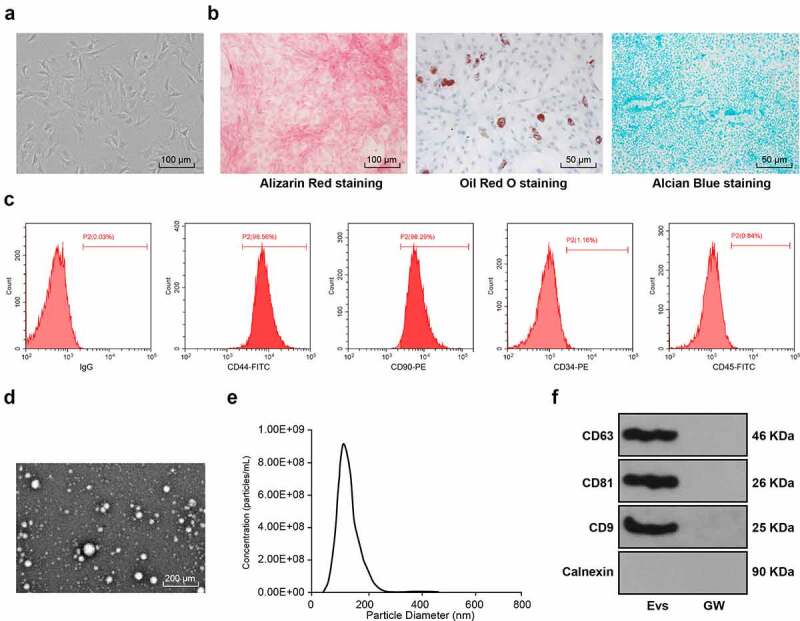
Identification of BMSC-EV. (a) Morphological observation of BMSCs under the microscope (100×); (b) The osteogenic, lipogenic, and chondrogenic differentiation characteristics of BMSCs were identified using Alizarin red, oil red O, and Alcian blue staining respectively; (c) flow cytometry detected BMSCs surface markers CD90, CD105, and CD34, CD45 expression; (d) Transmission electron microscopy of EV morphology; (e) qNano system for the detection of the size distribution of EVs; (f) Western blot analysis of expression of the EV positive markers CD63, CD81, CD9 and the negative marker Calnexin, using the supernatant of BMSC medium after GW4869 intervention as a control. The experiment was repeated three times independently

BMSCs isolated from rats were cultured to the P3 generation, and under the microscope the BMSCs were observed homogeneous, shuttle-shaped, and vortex-shaped (Figure 1a). The osteogenic, lipogenic, and chondrogenic differentiation characteristics of BMSCs were identified using Alizarin red, oil red O, and Alcian blue staining respectively, which revealed visible calcium junction deposits, lipid droplets, and acidic mucopolysaccharide accumulation in the cells (Figure 1b). Further detection of BMSCs surface antigen markers by flow cytometry showed that the expression rates of positive markers CD44 and CD90 were above 95% and the negative markers CD34 and CD45 did not exceed 2% (Figure 1c). The above results indicated that we successfully isolated and cultured rat BMSCs. Next, the EVs were isolated from BMSCs and identified, and the results showed isolated EVs were in a teletop-like bilayer structure (Figure 1d), and most of EVs had a particle size between 50 nm and 150 nm (Figure 1e). Western blot analysis detected the expression of the positive markers of CD63, CD81 and CD9 and did not detect the expression of the negative marker Calnexin. CD63, CD81 and CD9 were not significantly expressed in the supernatant of BMSCs medium after intervention with the EV generation inhibitor GW4869 (figure 1f), indicating that we successfully isolated BMSC-derived EVs.

### 3.2 BMSC-EVs improve motor dysfunction and tissue damage of hindlimbs in SCI rats

**Figure 2.**
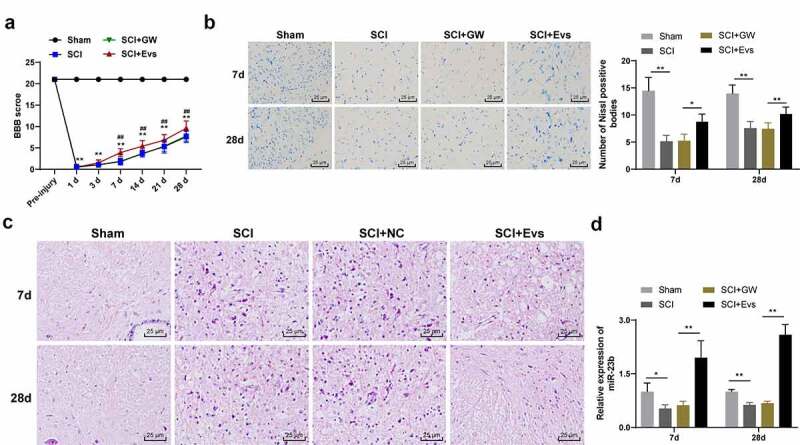
BMSC-EVs improve motor dysfunction and tissue damage of hindlimbs in SCI rats. (a) Motor recovery of hindlimbs of SCI rats assessed by BBB scoring at before modeling and days 1, 3, 7, 14, 21, and 28 after modeling (N = 24/12); (b) Nissl staining analysis of nerve cell injury in SCI rat spinal cord tissues on days 7 and 28 after modeling (200×); (c) HE staining analysis of SCI rat spinal cord histopathological changes on days 7 and 28 after modeling (200×); (d) RT-qPCR for expression of miR-23b in injured spinal cord tissue. n = 6. Data are expressed as mean ± standard deviation and analyzed using one-way ANOVA, with post hoc tests using Tukey’s multiple comparisons test, *p < 0.05, **p < 0.01, ##*p* < 0.01 (** indicates SCI group vs. sham group, ## indicates SCI+EVs group vs. SCI group)

MSCs-derived EVs carry a variety of bioactive substances such as mRNAs and miRNAs, which are important in the exchange of information between cells, and in the recovery of many diseases [33]. Thus, we supposed that BMSC-EVs are beneficial for SCI improvement. To confirm our hypothesis, we injected BMSC-EVs intravenously into rats to study their effects on SCI. BBB scores of rats in the SCI+GW, SCI and SCI+EVs groups were significantly decreased compared to the sham group. No significant differences were seen in the SCI+GW group compared to the SCI group on days 7, 14, 21, and 28 after modeling. The locomotor scores of rats in the SCI+EVs group were significantly increased (Figure 2a, all *p* < 0.01). Nissl staining and HE staining were performed on the spinal cord tissues of rats on days 7 and 28 after modeling. Compared with the sham group, the SCI, SCI+GW and SCI+EVs groups all had reductions in Nissl bodies; compared to the SCI group, no significant change was found in Nissl bodies in SCI+GW group, and significant increase was observed in Nissl bodies in SCI+EVs group (Figure 2b, all *p* < 0.01). HE staining showed that on the 7^th^ day after modeling, the spinal cord of rats in each group of SCI model had a disordered structure and a large amount of inflammatory cell infiltration compared with the sham group; compared with the SCI group, the SCI+EVs group, rather than the SCI+GW group, showed significant improvement in structure and inflammatory cell infiltration; on the 28th day after modeling, the improvement of spinal cord in SCI + EVs group was better (Figure 2c).

miR-23b could be involved in the repair process of SCI [26]. Our study found that miR-23b expression was clearly downregulated in the SCI group relative to the sham group (Figure 2d, *p* < 0.01); miR-23b expression in spinal cord tissues of SCI+GW group was not significantly different from that in the SCI group, but was upregulated in the SCI+EVs group (Figure 2d, *p* < 0.01). Therefore, we speculated that the upregulation of miR-23b after EVs intervention may improve SCI in rats.

### 3.3 EVs-miR-23b improves motor dysfunction and tissue damage of hindlimbs in SCI rats

**Figure 3.**
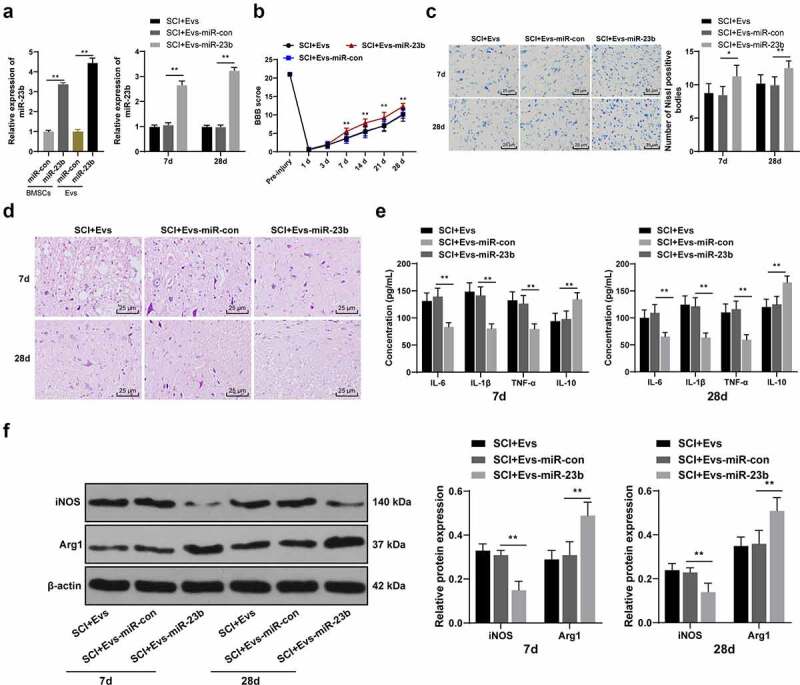
EV-miR-23b improves motor dysfunction and tissue damage of hindlimbs in SCI rats. (a) RT-qPCR analysis of the expression of miR-23b in BMSCs and EVs, and the expression of miR-23b in the spinal cord tissues of each group of rats on day 7 after modeling; (b) Motor recovery of hindlimbs of SCI rats assessed by BBB scoring at before modeling and days 7, 14, 21, and 28 after modeling (N = 24/12); (c) Nissl staining analysis of the neuronal damage in the spinal cord tissues of each group of rats on days 7 and 28 after modeling and the number of Nissl stained-neurons in the spinal cord; (d) HE staining of the pathological changes in the spinal cord of each group of rats on days 7 and 28 after modeling; (e) ELISA analysis of the protein content of IL-6, IL-1β, TNF-α and IL-10 in the spinal cord of each group of rats on days 7 and 28 after modeling; (f) Western blot analysis of the expression of iNOS and Arg1 in the spinal cord of each group of rats on days 7 and 28 after modeling. N = 6. Data are expressed as mean ± standard deviation, and analyzed using one-way ANOVA and Tukey’s multiple comparisons test, *p < 0.05, **p < 0.01

To further identify the role of miR-23b in EVs-enhanced SCI improvement, miR-23b mimic and its NC were transfected into BMSCs (BMSCs-miR-23b group and BMSCs-miR-con group), and miR-23b expression in BMSCs was examined. miR-23b expression was higher in the BMSCs-miR-23b group than that in the BMSCs-miR-con group (Figure 3a, *p* < 0.01). Also, miR-23b expression in EVs-miR-23b group was higher than that of EVs-miR-con group (Figure 3a, p < 0.01). The above results indicated the successful isolation of EVs from miR-23b-modified BMSCs.

We then injected EVs-miR-con and EVs-miR-23b intravenously 1 hour after the establishment of SCI in a rat model. miR-23b expression in spinal cord tissues was analyzed. Compared with the SCI+EVs-miR-con group, the SCI+EVs-miR-23b group on days 7 and 28 post-modeling showed significantly upregulated miR-23b expression (Figure 3a, *p* < 0.01). By assessing the behavioral performance of rats before modeling and on days 1, 3, 7, 14, 21, and 28 after modeling, we found compared to the EVs-miR-con group, EVs-miR-23b group from days 7, 14, 21, and 28 after modeling showed significantly improved motor ability of hindlimbs of SCI rats (Figure 3b, all *p* < 0.01). Nissl staining and HE staining were performed on the spinal cord tissues of rats on days 7 and 28 after modeling. Compared with the SCI+Evs-miR-con group, the SCI+Evs-miR-23b group had significantly increased Nissl bodies (Figure 3c, all *p* < 0.01). HE staining showed that compared with the SCI+Evs-miR-con group, the SCI+Evs-miR-23b group showed significant improvement in spinal structure and inflammatory cell infiltration (Figure 3d). Inflammatory response is an important cause of secondary injury after SCI [37]. Therefore, we further analyzed the effect of EVs-miR-23b on inflammatory response in spinal cord tissues. Compared with the SCI+EVs-miR-con group, the SCI+EVs-miR-23b group had significantly decreased levels of IL-6, IL −1β, and TNF-α and increased IL-10 (Figure 3e, all *p* < 0.01). The release of inflammatory factors in spinal cord tissues correlates with the differentiation of microglia, and microglia/macrophages have both M1 and M2 phenotypes, with the M1 phenotype promoting the release of pro-inflammatory factors and the M2 phenotype promoting the release of anti-inflammatory factors [35]. Expression of the microglial M1 phenotype marker iNOS and the M2 phenotype marker Arg1 was analyzed by western blot analysis (figure 3f). Compared to the SCI+EVs-miR-con group, EVs-miR-23b group exhibited significantly reduced iNOS expression and increased Arg1 expression (both *p* < 0.01). These results suggest that EVs-miR-23b is involved in the differentiation of microglia in injured spinal cord tissue, inhibit the inflammatory reaction and improve the recovery of injured tissue and motor ability.

### 3.4 EVs-miR-23b inhibits inflammation response in BV2 cells

**Figure 4.**
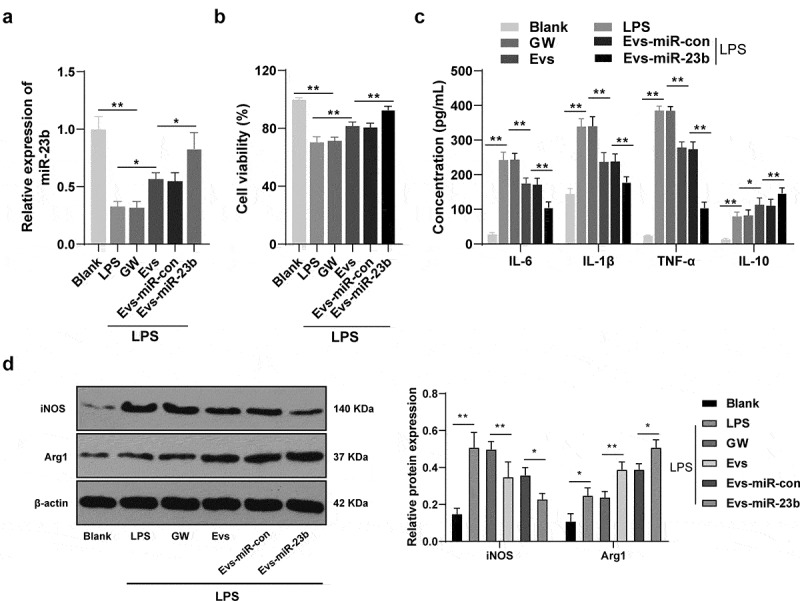
EV-miR-23b inhibits inflammation response in BV2 cells. (a) RT-qPCR analysis of miR-23b expression in BV2 cells; (b) MTT assay for BV2 cell viability; (c) ELISA for IL-6, IL1β, TNFα, and IL10 protein concentration in BV2 cells; (d) Western blot analysis for iNOS and Arg1 protein expression in BV2 cells. The cell experiment was repeated three times. Data are expressed as mean ± standard deviation and analyzed using one-way ANOVA and Tukey’s multiple comparisons test, *p < 0.05, **p < 0.01

To further investigate the mechanism of EVs-miR-23b, we then established a BV2 inflammatory cell model using LPS and incubated the cells with GW, EVs, EVs-miR-con and EVs-miR-23b for 24 hours, respectively. The expression of miR-23b was significantly increased in the EVs-miR-con group compared to the EVs-miR-23b group (Figure 4a, *p* < 0.05). MTT assay showed that LPS significantly reduced BV2 cell viability, and EVs treatment clearly increased cell viability; compared to that in the EVs-miR-con group, BV2 cell viability in the EVs-miR-23b group was significantly increased (Figure 4b, *p* < 0.01). Besides, LPS significantly increased IL-6, IL-1β, TNF-α and IL-10 in BV2 cells. The EVs group and EVs-miR-23b group showed significantly reduced IL-6, IL-1β, and TNF-α and increased IL-10 levels (Figure 4c, all *p* < 0.01). Western blot analysis of markers of M1 and M2 microglia in BV2 cells revealed that LPS significantly increased iNOS expression and reduced Arg1 expression; after EVs treatment, the expression of iNOS was decreased and the expression of Arg1 was increased; compared to the EVs-miR-con group, EVs-miR-23b group exhibited clearly decreased iNOS expression and increased Arg1 expression (Figure 4d, all *p* < 0.05). The above results were in general agreement with the results of animal experiments, indicating that EVs-miR-23b promoted microglial transformation to M2 type, inhibited microglial transformation to M1 type, and thereby inhibited inflammatory responses in cells in the LPS-induced microglial inflammation model.

### 3.5 EVs-miR-23b targets TLR4 and inactivates the NF-κB pathway

**Figure 5.**
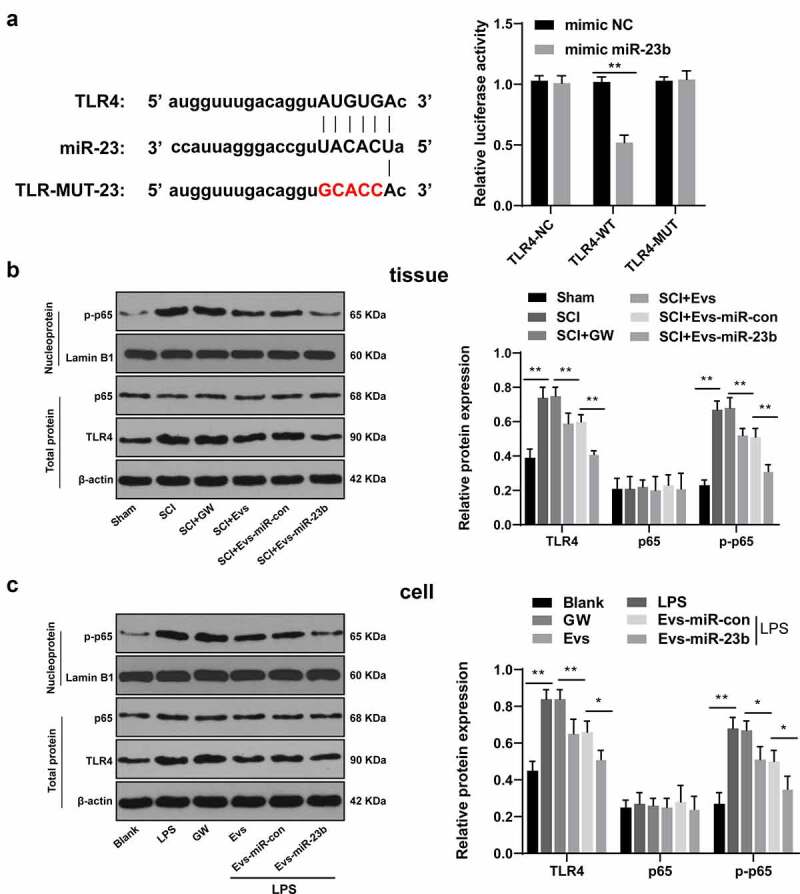
EV-miR-23b targets TLR4 and inactivates the NFκB pathway. (a) Dual luciferase detection of the targeting relationship between miR-23b and TLR4; (b) Western blot analysis of p65 and TLR4 protein levels in spinal cord tissues of rats and p-p65 level in nuclei on day 7 after SCI modeling, N = 6; (c) Western blot analysis of p65 and TLR4 protein levels in BV2 cells and p-p65 level in nuclei 24 hours after co-intervention of LPS and EVs. The cell experiment was repeated three times. Data are expressed as mean ± standard deviation, and comparisons among groups were analyzed using one-way ANOVA and Tukey’s multiple comparisons test, *p < 0.05, **p < 0.01

Activation of the TLR4/NF-kB pathway mediates the onset of priming inflammatory responses [38,39], so we speculated whether miR-23b could inhibit NF-kB activation by downregulating TLR4 expression to attenuate the inflammatory response. First, we focused on the binding sites between miR-23b and TLR4 via the Targetscan, and verified the binding relationship by dual-luciferase experiments (Figure 5a, *p* < 0.01). We further analyzed the levels of TLR4, p65 and p-p65 in the spinal cord on day 7 after modeling and in the LPS-induced BV2 inflammatory cell model. No significant differences were found in p65 expression in the spinal cord and cells after modeling, but TLR4 and p-p65 levels were obviously increased. The levels of TLR4 and p-p65 were decreased significantly after EVs treatment, and further decreased after EVs-miR-23b treatment (Figure 5b-C, all *p* < 0.05). All in all, EVs-miR-23b inhibited TLR4 expression and NF-kB activation.

### 3.6 Overexpression of TLR4 partially annuls the inhibitory effect of EVs-miR-23b on inflammatory response in SCI

**Figure 6.**
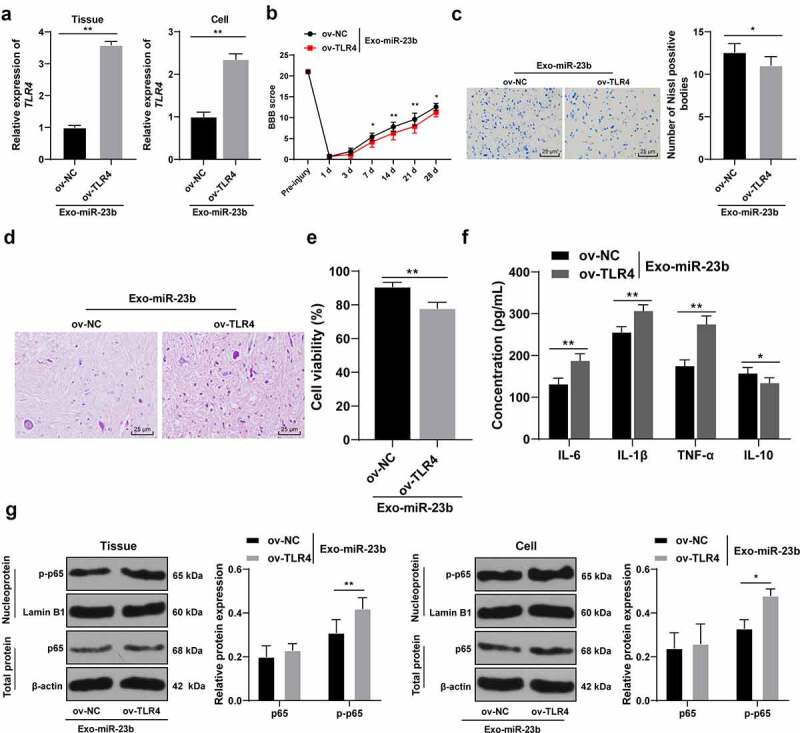
EV-miR-23b inhibits the TLR4/NFκB pathway activation in SCI. (a) RT-qPCR for the expression of TLR4 mRNA in rats and cells in the ov-NC+EVs-miR-23b and ov-TLR4+ EVs-miR-23b groups; (b) Motor recovery of hindlimbs of SCI rats assessed by BBB scoring at before modeling and days 1, 3, 7, 14, 21, and 28 after modeling (N = 12); (c) Nissl staining analysis of the neuronal damage in the spinal cord tissues of each group of rats on days 7 and 28 after modeling and the number of Nissl stained-neurons in the spinal cord; (d) HE staining of the pathological changes in the spinal cord of each group of rats on day 28 after modeling; (e) MTT assay for BV2 cell viability; (f) ELISA for changes in IL-6, IL-1β, TNF-α, and IL-10 protein content in each group; (g) Western blot analysis was used to detect the expression of p65 in spinal cord tissues and cells and p-p65 in nuclei. N = 6. The cell experiment was repeated three times. Data are expressed as mean ± standard deviation, and comparisons in figure A/C/E were analyzed using the t test, and comparisons in figure B/F/G/H were analyzed using one-way ANOVA and Tukey’s multiple comparisons test, *p < 0.05, **p < 0.01

To further validate that EVs-miR-23b can affect NF-kB activation by regulating the TLR4 expression, we constructed the TLR4 lentiviral overexpression vector ov-TLR4 and its control ov-NC. In the *in vivo* and *in vitro* model, TLR4 function rescue experiment was carried out. Compared to the ov-NC+EVs-miR-23b group, the ov-TLR4+ EVs-miR-23b group significantly elevated TLR4 mRNA expression (Figure 6a, p < 0.01). BBB score showed that TLR4 overexpression significantly reduced the motor ability of lower limbs of SCI rats (Figure 6b, *p* < 0.05). Nissl staining and HE staining were performed on the spinal cord tissue on the 28^th^ day after modeling. After overexpression of TLR4, the number of Nissl bodies was significantly reduced (Figure 6c, *p* < 0.05), and the spinal cord structure disorder and inflammatory cell infiltration were significantly aggravated (Figure 6d). The MTT results showed that compared with the ov-NC+EVs-miR-23b, the cell viability in the ov-TLR4+ EVs-miR-23b group was significantly decreased (Figure 6e, *p* < 0.01), and ELISA revealed that the content of IL-6, IL-1β, and TNF-α in the ov-TLR4+ EVs-miR-23b group were increased and IL-10 was reduced (figure 6f, all *p* < 0.05). In addition, after overexpression of TLR4, the expression of p65 remained unchanged, but the expression of p-p65 was significantly increased (Figure 6g, all *p* < 0.05)

## 4. Discussion

MSCs-EVs carry a variety of mRNAs and miRNAs and play pivotal roles in the information exchange between cells and in the recovery process of many diseases [33]. It is reported that inhibiting NF-κB Pathway can improve nerve injury through the anti-inflammatory effects [40,41]. A study has pointed out that the downregulation of NEAT1 can reduce the inflammatory response through the miR-211-5p/MAPK1 axis to improve SCI [42]. This study explored the potential molecular mechanisms underlying the therapeutic potential of miR-23b-modified BMSCs-EVs for the improvement of SCI. We ultimately highlighted that miR-23b delivered by BMSC-EVs targeted TLR4 and inhibited the activation of NF-κB pathway, thus improving lower limb activity, tissue injury and inflammatory reaction in SCI rats.

Mesenchymal derived EVs are reported to facilitate motor function recovery in a monkey model of cortical injury [43]. The current study revealed that the locomotor scores and Nissl bodies were significantly increased and structure and inflammatory cell infiltration were ameliorated in rats in the SCI+EVs group. BMSC-exosomes decreased neural behavioral scores and inflammatory cell infiltration in experimental autoimmune encephalomyelitis rats by regulating the polarization of microglia [44]. Similarly, the miR-29b exosome treatment exhibited significantly higher BBB scores (5–16 points) at 1, 2, 4, and 8 weeks post-injection than that in SCI group (0–6 points) [45]. In brief, BMSC-EVs improved motor dysfunction and tissue damage of hindlimbs in SCI rats.

A combination of miRNAs and neuron-derived exosomes may be a promising minimally invasive approach for the treatment of SCI [46]. miR-23b is involved in the repair process of SCI [26]. We further identified miR-23b expression was clearly downregulated in the SCI group and upregulated in the SCI+EVs group. To further verify the role of miR-23b in the promoting effects of EVs on SCI improvement, miR-23b mimic and its NC were transfected into BMSCs, and then EVs were extracted. From days 7, 14, 21 and 28 after modeling, EVs-miR-23b treatment significantly improved motor ability of hindlimbs of SCI rats, increased Nissl bodies, improved spinal structure and inflammatory cell infiltration. Similarly, injection of miR-29b exosomes alleviated histopathological damage and promoted neuronal regeneration in spinal cord tissues of SCI rats [45]. Inflammatory response is a main cause of secondary injury after SCI [37]. In SCI, elevated levels of IL-6 are known to correlate with systemic inflammation [47]. We elicited that EVs-miR-23b treatment also decreased levels of IL-6, IL-1β, and TNF-α and increased IL-10. miR-23b-infusion in neuropathic pain-induced animals significantly improved motor ability [26]. Moreover, the release of inflammatory factors in spinal cord tissues correlates with the differentiation of microglia [35]. Microglia are critical in neuroinflammation following CNS injury [9]. M1-like phenotypes increase secretion of pro-inflammatory mediators and exacerbate injury [9]. M2-like phenotypes have anti-inflammatory and neurorestorative effects following SCI [48]. Neuron-derived exosomes-transmitted miR-124-3p protect traumatically injured spinal cord by suppressing the activation of neurotoxic microglia [46]. Our results revealed that EVs-miR-23b treatment reduced iNOS expression and increased Arg1 expression. These results indicated that EVs-miR-23b regulated the differentiation of microglia after SCI, inhibited inflammatory responses, and improved recovery of motor abilities. Furthermore, the results in the BV2 inflammatory cell model using LPS were in general agreement with those in animal experiments. miR-23b inhibited the levels of inflammatory factors in LPS-stimulated vascular endothelial cells [49]. miR-23b attenuated the HO-induced injury of microglial cells via TAB3/NF-κB pathway in an *in vitro* model of SCI [25]. In summary, EVs-miR-23b promoted microglial transformation to M2 type, thereby inhibiting inflammatory responses in LPS-induced microglial inflammation model.

miR-23b attenuated the microglia apoptosis through suppressing NF-κB activation in SCI models [25]. TLR4/NF-κB axis is involved in SCI [50]. TLR4 expression was enhanced in serum samples of patients with SCI [51]. BBB score was negatively correlated with TLR4 and NF-κB levels [52]. It is reported that neuroinflammation plays a crucial role in the second stage of SCI, which begins after TLR4 activation [53]. Sansing et al. showed that activated microglia highly expressed TLR4, which is involved the determination of microglia phenotype and function and induced inflammatory injury in central nervous system lesions [54]. Microglia are the most commonly expressed cell type of TLR4 [55,56]. The activation of microglia may mediate the early inflammatory response of SCI through TLR4 and its downstream signal pathway. Therefore, we speculated that miR-23b could regulate the NF-kB/TLR4 to ameliorate inflammatory response in SCI. In addition, miR-23b-5p has a binding relationship with TLR4 [57]. We predicted the binding sites of miR-23b and TLR4 by Targetscan database analysis, and verified the binding relationship between miR-23b and TLR4 by dual-luciferase experiment. At present, the mechanism of miR-23b binding to TLR4 in SCI has not been reported. EVs-miR-23b treatment significantly decreased levels of TLR4 and p-p65. BMSC-EV reduced brain cell death, enhanced neuronal survival and regeneration, and improved motor function after SCI via downregulation of NF-κB p65 signaling [14]. To further validate that EVs-miR-23b can affect NF-kB activation by regulating TLR4, we constructed ov-TLR4 and ov-NC vectors. The cell viability in the ov-TLR4+ EVs-miR-23b group was significantly decreased, and the content of IL-6, IL-1β, and TNF-α were increased and IL-10 was reduced. Similarly, overexpression of TLR4 reversed the inhibitory effects of miR-182-5p overexpression on inflammation and apoptosis in SCI [58]. MSC-exosome-shuttled miR-216a-5p promoted functional behavioral recovery after SCI in mice by shifting microglial M1/M2 polarization via the TLR4/NF-κB/PI3K/AKT cascade [35]. Microglia are neuroimmune cells that participate in the maintenance of the environment in the central nervous system and respond to injury and repair. They are in a “resting state” under normal conditions. After activation, they carry out phenotypic and functional transformation and maintain tissue homeostasis [59–61]. The activation of microglia plays a key role in regulating inflammation and immune response. Activated microglia are divided into M1 (pro-inflammatory) and M2 (anti-inflammatory) microglia according to different phenotypes [62]. LPS induces microglia activation. On the one hand, it upregulates the expression of M1 polarization marker iNOS, which polarizes microglia to M1 type, that is, pro-inflammatory phenotype, and promotes inflammation and neuronal damage; On the other hand, M1 microglia transform into M2, that is, anti-inflammatory phenotype, and the expression of M2 polarization marker Arg1 on the cell surface is increased, which plays an anti-inflammatory role and promotes the recovery of neural function. They are considered as nerve repair cells [62–65]. These results suggest that EVs-miR-23b can inhibit TLR4 expression and NF KB activation, and overexpression of TLR4 partially reverses the inhibitory effect of EVs-miR-23b on SCI.

## 5. Conclusion

In summary, miR-23b delivered by BMSC-EVs could improve SCI in rats by targeting TLR4 and inhibiting the activation of NF-κB pathway. These results discovered a novel theoretical option for SCI recovery. Still, this is just a preclinical research, and the experiment results and effective application into clinical practice need further validation. In the future, we will further probe the underlying mechanisms of other targets of miR-23b. More attention will be paid to seek reliable therapies for SCI recovery.

## Data Availability

All the data generated or analyzed during this study are included in this published article.
